# Outcomes of multi‐hole self‐expandable metal stents versus fully covered self‐expandable metal stents for malignant distal biliary obstruction in unresectable pancreatic cancer

**DOI:** 10.1002/deo2.70014

**Published:** 2024-09-24

**Authors:** Tsuyoshi Takeda, Takashi Sasaki, Takeshi Okamoto, Takafumi Mie, Yoichiro Sato, Yuri Maegawa, Tatsuki Hirai, Yukari Suzuki, Takaaki Furukawa, Masato Ozaka, Naoki Sasahira

**Affiliations:** ^1^ Department of Hepato‐Biliary‐Pancreatic Medicine Cancer Institute Hospital of Japanese Foundation for Cancer Research Tokyo Japan

**Keywords:** covered self‐expandable metal stent, endoscopic biliary drainage, endoscopic biliary stenting, malignant distal biliary obstruction, pancreatic cancer

## Abstract

**Objectives:**

The multi‐hole self‐expandable metal stent (MHSEMS) is a novel SEMS with multiple small side holes on the covering membrane to prevent stent migration while minimizing tumor ingrowth. This study aimed to evaluate the clinical outcomes of MHSEMS in comparison with conventional covered SEMS (c‐CMS).

**Methods:**

Consecutive patients with unresectable pancreatic cancer who underwent initial SEMS placement (MHSEMS or c‐CMS) for malignant distal biliary obstruction were analyzed. Technical success, clinical success, causes of recurrent biliary obstruction (RBO), non‐RBO adverse events, time to RBO (TRBO), and endoscopic reintervention were compared between groups.

**Results:**

A total of 65 patients were included (MHSEMS: 27, c‐CMS: 38). The technical success, clinical success, and non‐RBO adverse event rates were similar between groups. Although stent migration was less frequently observed in the MHSEMS group (0% vs. 17.6%, *p* = 0.032), overall RBO rates were similar between groups (53.8% vs. 55.9%, *p* > 0.99). The most common cause of RBO within 14 days in the MHSEMS group was non‐occlusion cholangitis. Median TRBO was significantly shorter in the MHSEMS group (101 vs. 227 days, *p* = 0.030) and MHSEMS was an independent predictor for shorter TRBO in multivariate analysis (hazard ratio, 2.27; 95% confidence interval, 1.06–4.86; *p* = 0.034). Outcomes after endoscopic interventio were not significantly different between groups. Stent removal was successful in all attempted cases in both groups.

**Conclusions:**

MHSEMS was associated with a significantly shorter TRBO compared to c‐CMS. Further modifications of the present MHSEMS may be needed.

## INTRODUCTION

Endoscopic biliary drainage is the standard of care for malignant distal biliary obstruction (MDBO). Self‐expandable metal stents (SEMSs) are preferred over plastic stents for the palliation of unresectable MDBO due to their longer stent patency and fewer re‐intervention rates.^1^, ^2^ Conventional covered SEMSs (c‐CMSs), which were developed to prevent stent occlusion resulting from tumor ingrowth, have been reported to reduce recurrent biliary obstruction (RBO) relative to uncovered SEMSs, especially in patients with pancreatic cancer (PC).^3^ Stent migration remains a significant problem associated with c‐CMSs, although anti‐migration properties have contributed to the reduction of c‐CMS migration.^4^, ^5^

Multi‐hole SEMS (MHSEMS) is a novel type of SEMS that has multiple side holes along the covering membrane. This stent was originally developed to prevent obstruction of side branches when used in malignant hilar biliary obstruction,^6^, ^7^ but was also expected to be useful in MDBO by preventing stent migration by taking advantage of minimal tumor ingrowth.^6^ A retrospective analysis showed that MHSEMS achieved longer stent patency and lower RBO rates compared to uncovered SEMS, while the outcomes were not statistically significant when compared to fully covered SEMS.^8^ Thus, the superiority of MHSEMS over fully covered SEMS has not been fully elucidated.

Therefore, we conducted this retrospective study to evaluate the efficacy and safety of MHSEMSs in comparison with c‐CMSs as the initial SEMS in patients with unresectable PC.

## METHODS

### Patients

Consecutive patients with unresectable PC who underwent initial SEMS (MHSEMS or c‐CMS) placement for MDBO at our institution between October 2020 and December 2023 were identified from our prospectively maintained database. Excluded patients were as follows: (1) patients with SEMS placed above the papilla, (2) patients with surgically altered anatomy, and (3) patients with concomitant hilar biliary obstruction. Stent selection was mainly determined by the time period in which the SEMSs were placed. In general, c‐CMSs were used between October 2020 and November 2022, while MHSEMSs were used between December 2022 and December 2023. This study was conducted in accordance with the master protocol (2023‐GB‐077) for gastrointestinal, hepatic, and pancreatobiliary diseases, which was approved by the institutional review board of our institution. Informed consent for this study was waived by the institutional review board owing to the retrospective study design. The study was publicized on the hospital website, allowing patients to opt out of this study for any reason.

### The design of SEMS

**FIGURE 1:**
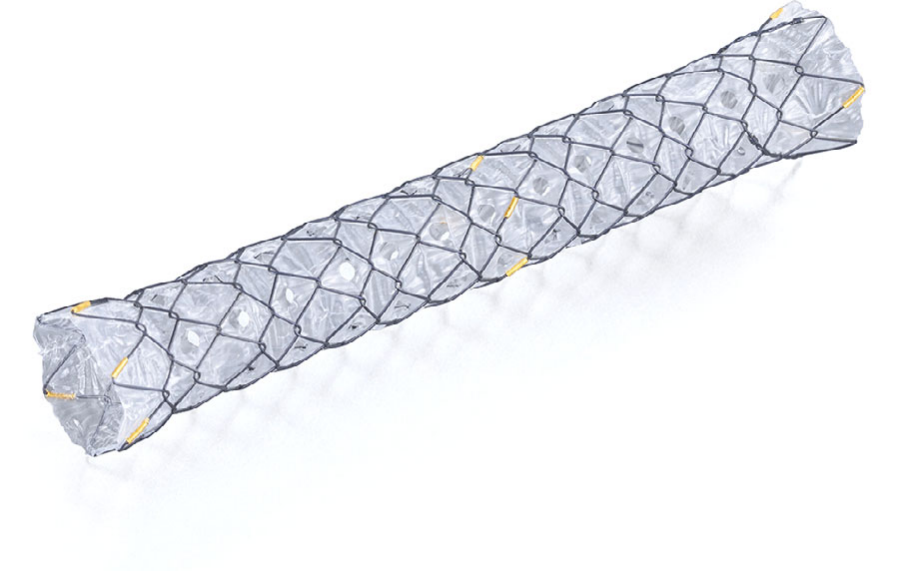
The multi‐hole self‐expandable metal stent.

The MHSEMS used in this study was a fully covered SEMS with multiple side holes on the covering membrane (HANAROSTENT Biliary Multi‐hole NEO; M.I.Tech; Figure 1). The stent is made of nitinol wire and is covered with a silicon membrane, whose body structure is the same as the c‐CMS used in this study (described below). The stent was designed to prevent stent migration through small tissue ingrowths that form in the multiple small side holes (1.8 mm) along the covering membrane. The delivery system is 8 Fr in diameter. All patients received MHSEMSs with diameters of 10 mm and lengths of 6, 7, or 8 cm. The 6, 7, and 8 cm stents have 66, 78, and 90 holes, respectively, arranged in six columns spaced evenly around the stent.

The c‐CMS used in this study was a fully covered SEMS (HANAROSTENT Biliary Full Cover NEO; M.I.Tech). All patients received SEMSs with diameters of 10 mm and lengths of 6, 7, or 8 cm.

### Endoscopic procedures

Endoscopic retrograde cholangiopancreatography (ERCP) was performed using a therapeutic duodenoscope (JF260, TJF260, TJF‐Q290V; Olympus Medical Systems) under conscious sedation. More than half of the patients received previous biliary drainage with plastic stents (mainly at the referring hospital) or endoscopic nasobiliary drainage (ENBD) tubes (mainly due to an unconfirmed diagnosis of PC or cholangitis) before SEMS placement. Endoscopic sphincterotomy was performed in all patients. Placement of prophylactic pancreatic stent and administration of prophylactic rectal nonsteroidal anti‐inflammatory drugs (NSAIDs) were conducted at the treating endoscopist's discretion. The length of SEMS was determined based on cholangiographic findings. All procedures were performed by experienced pancreaticobiliary endoscopists or by trainees under their direct supervision.

### Outcome measurements

Clinical outcomes of endoscopic biliary drainage were generally assessed according to the revised Tokyo Criteria 2024.^9^ The primary outcome was time to RBO (TRBO), defined as the time between stent placement and biliary drainage or stent removal for RBO. Patients who were lost to follow‐up or alive at the end of the study period, underwent stent removal due to non‐RBO adverse events (AEs), underwent conversion surgery, or died without RBO were treated as censored cases on the date of the last follow‐up, stent removal, conversion surgery, or death, respectively. Asymptomatic stent migration was not treated as a censoring event, while symptomatic stent migration was treated as RBO. The secondary outcomes were technical success, clinical success, causes of RBO, AEs, overall survival (OS), and endoscopic reintervention (ERI). Technical success was defined as successful SEMS placement in the intended location. Clinical success was defined as a ≥50% reduction or normalization of total bilirubin (for cases with jaundice), a ≥50% reduction or normalization of target liver enzymes (for cases undergoing drainage for elevated levels of other liver enzymes), or resolution of cholangitis (for cases undergoing drainage for cholangitis without jaundice), within 14 days of the index biliary drainage. If cholangitis had resolved at the time of SEMS placement due to preceding ENBD, clinical success was defined as no exacerbation of cholangitis after SEMS placement. RBO was defined as a composite endpoint of stent occlusion or migration requiring biliary drainage or stent removal, evaluated only in cases with technically and clinically successful SEMS placement. AEs were defined as any event requiring treatment in addition to RBO and were classified as RBO and non‐RBO AEs. Non‐occlusion cholangitis was treated as RBO when endoscopic biliary drainage was required, while it was treated as non‐RBO AEs when it improved with antibiotics without requiring any endoscopic interventions. The severity of non‐RBO AEs was graded according to the American Society of Gastrointestinal Endoscopy lexicon guidelines.^10^ Duodenal invasion was diagnosed based on endoscopic findings at the index biliary drainage. The amount of ascites was evaluated using the most recent computed tomography scan before SEMS placement and was categorized according to the Japanese Classification of Gastric Carcinoma:^11^ none, ascites undetected by computed tomography; mild, ascites localized in only one area such as the pelvic cavity; moderate, ascites neither mild nor severe; and severe, ascites throughout the abdominal cavity. The diameter of the SEMS at the level of the biliary stricture was measured using an abdominal X‐ray taken on the following day. Follow‐up data was confirmed until June 30, 2024.

### Statistical analysis

Categorical variables are described as numbers with proportions and were compared using the Chi‐square test or Fisher's exact test as appropriate. Continuous variables are expressed as medians with ranges and were compared using the Mann‐Whitney U test. TRBO and OS were estimated using the Kaplan‐Meier product‐limit method and were compared using the log‐rank test. The Cox proportional hazards model was used to identify risk factors for TRBO. Factors with P values < 0.20 were considered to be potential risk factors and were included in multivariate analysis. The median time to each type of RBO was calculated as the median time to RBO for those who developed the event. *p‐*Values < 0.05 were considered statistically significant. All statistical analyses were carried out using the EZR software version 1.40.^12^

## RESULTS

### Patient characteristics

A total of 65 patients were included (MHSEMS: 27; c‐CMS: 38). Baseline and procedural characteristics are summarized in Table 1. A higher proportion of patients received prophylactic rectal NSAIDs in the MHSEMS group (81.5% vs. 57.9%, *p* = 0.061), although the difference was not statistically significant. The diameter of SEMS at the level of the biliary stricture, measured on the following day, was smaller in those who received MHSEMS (5.6 mm vs. 6.2 mm, *p* = 0.10). Other baseline and procedural characteristics were similar between the two groups.

**TABLE 1:** Baseline and procedural characteristics.

	MHSEMS, *n* = 27	c‐CMS, *n* = 38	*p*‐value
Age, years	67 (46–82)	67 (53–86)	0.58
Sex, male	15 (55.6%)	20 (52.7%)	>0.99
ECOG performance status 0/1/2–3	15 (55.6%)/ 10 (37.0%)/ 2 (7.4%)	23 (60.6%)/ 11 (28.9%)/ 4 (10.5%)	0.75
History of cholecystectomy	1 (3.7%)	0 (0%)	0.42
Tumor status			0.79
Locally advanced	9 (33.3%)	11 (28.9%)	
Metastatic	18 (66.7%)	27 (71.1%)	
Duodenal invasion	5 (18.5%)	9 (23.7%)	0.76
Co‐existing duodenal metal stent	1 (3.7%)	2 (5.3%)	>0.99
Moderate to severe ascites	1 (3.7%)	5 (13.2%)	0.39
Peritoneal dissemination	4 (14.8%)	11 (28.9%)	0.24
Tumor involvement of orifice of cystic duct^†^	3 (11.5%)	3 (7.9%)	0.68
Tumor involvement of the pancreatic duct	24 (88.9%)	34 (89.5%)	>0.99
Previous biliary drainage	17 (63.0%)	29 (76.3%)	0.28
Previous gallbladder drainage^‡^	1 (3.7%)	2 (5.3%)	>0.99
Chemotherapy after SEMS placement	23 (85.2%)	31 (81.6%)	0.75
Stent diameter, 10 mm	27 (100%)	38 (100%)	>0.99
Stent length, 6/7/8 cm	6 (22.2%)/ 13 (48.2%)/ 8 (29.6%)	11 (28.9%)/ 20 (52.7%)/ 7 (18.4%)	0.64
Endoscopic sphincterotomy	27 (100%)	38 (100%)	>0.99
Prophylactic pancreatic stent	1 (3.7%)	0 (0%)	0.42
Prophylactic rectal NSAIDs use	22 (81.5%)	22 (57.9%)	0.061
Diameter of SEMS at the level of the biliary stricture^¶^, mm	5.6 (4.2–9.0)	6.2 (4.2–10.0)	0.10

Continuous variables are expressed as median (range) and categorical variables are expressed as absolute numbers (proportions).

^†^ Denominators adjusted to exclude one patient who underwent cholecystectomy in the MHSEMS group.

^‡^ Three patients had undergone gallbladder drainage due to cholecystitis before SEMS placement. Methods of gallbladder drainage were endoscopic gallbladder stenting in two patients and endoscopic ultrasound‐guided gallbladder drainage in one patient.

^¶^ Diameter of the SEMS at the level of the biliary stricture was measured using an abdominal X‐ray taken on the following day.

Abbreviations: c‐CMS, conventional covered metal stent; ECOG, Eastern Cooperative Oncology Group; MHSEMS, multi‐hole self‐expandable metal stent; NSAIDs, non‐steroidal anti‐inflammatory drugs; SEMS, self‐expandable metal stent.

### Clinical outcomes of SEMS

The outcomes of SEMSs are described in Table 2. There were no significant differences in technical success rates and clinical success rates (96.3% vs. 89.5%, *p* = 0.39) between the two groups. The total AE rates (RBO and non‐RBO AEs) were also similar between the two groups (63.0% vs. 65.8%, *p* > 0.99).

**TABLE 2:** Outcomes of initial self‐expandable metal stent (SEMS) placement.

	MHSEMS, *n* = 27	c‐CMS, *n* = 38	*p*‐value
Technical success	27 (100%)	38 (100%)	>0.99
Clinical success	26 (96.3%)	34 (89.5%)	0.39
Adverse events	17 (63.0%)	25 (65.8%)	>0.99
Non‐RBO adverse events	3 (11.1%)	8 (21.1%)	0.34
Pancreatitis	1 (3.7%)	6 (13.2%)	0.22
Mild/moderate/severe	0/ 1/ 0	2/ 4/ 0	
Cholecystitis^†^	0 (0%)	2 (5.6%)	0.51
Mild/moderate/severe		0/ 2/ 0	
Non‐occlusion cholangitis	1 (3.7%)	0 (0%)	0.42
Mild/moderate/severe	1/ 0/ 0		
Bleeding	1 (3.7%)	0 (0%)	0.42
Mild/moderate/severe	0/ 0/ 1		
RBO^‡^	14 (53.8%)	19 (55.9%)	>0.99
Occlusion	8 (30.8%)	9 (26.5%)	0.78
Biliary debris/stones	4	9	
Food impaction	1	0	
Tumor ingrowth	0	0	
Tumor overgrowth	0	0	
Hemorrhage	2	0	
Kinking	1	0	
Migration	0 (0%)	6 (17.6%)	0.032
Proximal migration	0	1	
Distal migration	0	5	
Complete migration	0	5	
Incomplete migration	0	0	
Non‐occlusion cholangitis	6 (23.1%)	4 (11.8%)	0.31
Median time to each type of RBO^¶^, days			
Occlusion	73	143	0.092
Migration	Not available	287	
Non‐occlusion cholangitis	13	160	0.010

Categorical variables are expressed as absolute numbers (proportions).

^†^ Denominators adjusted to exclude four patients who had a history of cholecystectomy or gallbladder drainage before SEMS placement (two patients in each group).

^‡^ Denominators adjusted to exclude five patients who did not achieve clinical success (one patient and four patients in the MHSEMS and c‐CMS groups, respectively).

^¶^ Median time to each type of RBO was calculated as the median time to RBO for those who developed the event.

Abbreviations: c‐CMS, conventional covered metal stent; MHSEMS, multi‐hole self‐expandable metal stent; RBO, recurrent biliary obstruction; SEMS, self‐expandable metal stent.

There were no significant differences in non‐RBO AE rates (11.1% vs. 21.1%, *p* = 0.34) or in any specific type of non‐RBO AEs including pancreatitis (3.7% vs. 13.2%, *p* = 0.22), and cholecystitis (0% vs. 5.6%, *p* = 0.51) between the two groups. Of the seven patients who developed pancreatitis, stent removal was performed on the following day in four (MHSEMS: 1; c‐CMS: 3). Of the two patients who developed cholecystitis, percutaneous transhepatic gallbladder aspiration was performed in one, and percutaneous transhepatic gallbladder drainage followed by EUS‐guided gallbladder drainage in the other. One patient in the MHSEMS group underwent transcatheter arterial embolization for bleeding due to cystic artery pseudoaneurysm.^13^

Although stent migration was less frequently observed in the MHSEMS group (0% vs. 17.6%, *p* = 0.032), overall RBO rates were not significantly different between the two groups (53.8% vs. 55.9%, *p* > 0.99). The most common cause of RBO within 14 days in the MHSEMS group was non‐occlusion cholangitis, and the median time to non‐occlusion cholangitis was significantly shorter in the MHSEMS group (13 vs. 160 days, *p* = 0.010).

**FIGURE 2:**
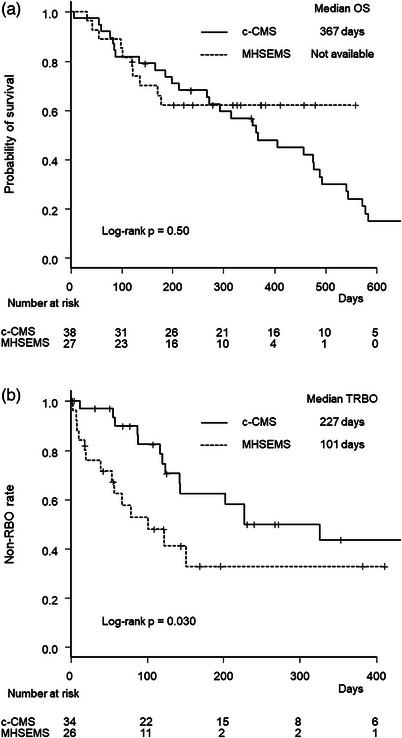
Kaplan‐Meier curves by stent group. (a) Overall survival. (b) Time to recurrent biliary obstruction. MHSEMS, multi‐hole self‐expandable metal stent; c‐CMS, conventional covered metal stent; OS, overall survival; RBO, recurrent biliary obstruction; TRBO, time to recurrent biliary obstruction.

Kaplan‐Meier curves of OS and TRBO are illustrated in Figure 2. Median OS was not significantly different between the two groups (not available vs. 367 days, *p* = 0.50). Median TRBO was significantly shorter in the MHSEMS group (101 vs. 227 days, *p* = 0.030). The non‐RBO rates at 3, 6, and 12 months were 53%, 33%, and 33%, respectively, in the MHSEMS group, and 82%, 62%, and 44%, respectively, in the c‐CMS group.

Multivariate analysis identified MHSEMS (hazard ratio, 2.27; 95% confidence interval, 1.06–4.86; *p* = 0.034) as an independent predictor of shorter TRBO (Table 3).

**TABLE 3:** Univariate and multivariate analyses for time to recurrent biliary obstruction.

		Univariate				Multivariate	
		**HR**	**95% CI**	***p*‐value**	**HR**	**95% CI**	***p*‐value**
Age	>70 years	1.65	0.81–3.36	0.17	1.66	0.81–3.38	0.17
Sex	Male	0.77	0.38–1.53	0.45			
ECOG PS	0	0.76	0.35–1.65	0.49			
Tumor status	Metastatic	1.16	0.55–2.45	0.69			
Co‐existing duodenal metal stent	Yes	0.55	0.12–2.45	0.44			
Duodenal invasion	Yes	0.57	0.23–1.38	0.21			
Ascites, moderate to severe	Yes	2.60	0.60–11.3	0.20			
Peritoneal dissemination	Yes	1.61	0.76–3.42	0.21			
Previous biliary drainage	Yes	1.30	0.58–2.89	0.52			
Type of SEMS	MHSEMS	2.27	1.06–4.85	0.035	2.27	1.06–4.86	0.034

Abbreviations: CI, confidence interval; ECOG, Eastern Cooperative Oncology Group; HR, hazard ratio; MHSEMS, multi‐hole self‐expandable metal stent; PS, performance status; SEMS, self‐expandable metal stent.

### ERI after RBO

Outcomes of ERI after the first RBO are summarized in Table 4. Technical and clinical success rates of ERI were similar between the two groups. There was one case of ERI failure due to difficulty in passing the duodenoscope through the duodenal stricture in each group. Of these, the patient in the MHSEMS group underwent EUS‐guided hepaticogastrostomy and the patient in the c‐CMS group underwent percutaneous transhepatic biliary drainage followed by percutaneous anterograde SEMS placement. Stent removal was successful in all attempted cases in both groups. Stent removal was not attempted in six cases due to duodenal stricture (MHSEMS: 1), hemobilia (MHSEMS: 1), or based on the endoscopists’ discretion (MHSEMS: 1; c‐CMS: 3). The final drainage methods were plastic stent placement (MHSEMS: 1; c‐CMS: 4), SEMS placement (MHSEMS: 8; c‐CMS: 11), removal of biliary debris/stones or food residues (MHSEMS: 1; c‐CMS: 2), stent‐free (MHSEMS: 1), EUS‐guided hepaticogastrostomy (MHSEMS: 2; c‐CMS: 2), and EUS‐guided choledochoduodenostomy (MHSEMS: 1). RBO rates (53.8% vs. 44.4%, *p* = 0.72) and median TRBO (111 vs. 216 days, *p* = 0.47) after ERI were not significantly different between the two groups.

**TABLE 4:** Outcomes of endoscopic reintervention after the first recurrent biliary obstruction.

	MHSEMS, *n* = 14	c‐CMS, *n* = 19	*p*‐value
Technical success	13 (92.9%)	18 (94.7%)	>0.99
Clinical success	13 (92.9%)	18 (94.7%)	>0.99
Patients with indwelling SEMS^†^	14 (100%)	14 (73.7%)	0.054
Successful stent removal in attempted cases^‡^	11/11 (100%)	11/11 (100%)	>0.99
Indwelling period of SEMS in attempted cases^‡^	20 (3–122)	227 (12–533)	0.001
Final drainage method			
Plastic stent	1 (7.1%)	4 (21.1%)	
SEMS^§^	8 (57.3%)	11 (57.9%)	
Removal of biliary debris/stones or food residues	1 (7.1%)	2 (10.5%)	
Stent‐free	1 (7.1%)	0	
EUS‐guided hepaticogastrostomy	2 (14.3%)	2 (10.5%)	
EUS‐guided choledochoduodenostomy	1 (7.1%)	0	
RBO after reintervention	7 (53.8)	8 (44.4)	0.72
Median TRBO after reintervention, days	111	216	0.47

Continuous variables are expressed as median (range) and categorical variables are expressed as absolute numbers (proportions).

^†^ Complete distal migration occurred in five patients in the c‐CMS group.

^‡^ Stent removal was not attempted in six patients (three patients in each group).

^§^ Two patients underwent SEMS placement in a stent in the stent method (one patient in each group) and one patient underwent SEMS placement via a percutaneous transhepatic biliary drainage route.

Abbreviations: c‐CMS, conventional covered metal stent; ENBD, endoscopic nasobiliary drainage; EUS, endoscopic ultrasound; MHSEMS, multi‐hole self‐expandable metal stent; RBO, recurrent biliary obstruction; SEMS, self‐expandable metal stent; TRBO, time to recurrent biliary obstruction.

## DISCUSSION

In this retrospective study of unresectable PC patients with DMBO, we evaluated the clinical outcomes of MHSEMSs in comparison with c‐CMSs using the revised Tokyo Criteria 2024.^9^ We found that MHSEMSs were associated with a significantly shorter TRBO compared to c‐CMSs. Although stent migration was not observed in the MHSEMS group, overall RBO rates were not different between the two groups. Other clinical outcomes including total AE rates, non‐RBO AE rates, and outcomes after ERI were also not different between the two groups. As the body structure of the two stents was identical except for the presence of multiple side holes, this study highlights the need for further modification of the present MHSEMS.

Endoscopic biliary drainage using SEMS is the mainstay for the treatment of unresectable DMBO. The causes of RBO are different between uncovered SEMSs and c‐CMSs. Uncovered SEMSs are subject to tumor ingrowth, while c‐CMSs are prone to stent migration. The MHSEMS was designed to overcome these two disadvantages by adding multiple small side holes on the covering membrane.^6^, ^7^ The covering membrane is suspected to prevent complete tumor ingrowth, while the small side holes are suspected to prevent stent migration due to small ingrowths. Therefore, MHSEMSs may theoretically be the ideal type of SEMS for unresectable DMBO.

**FIGURE 3:**
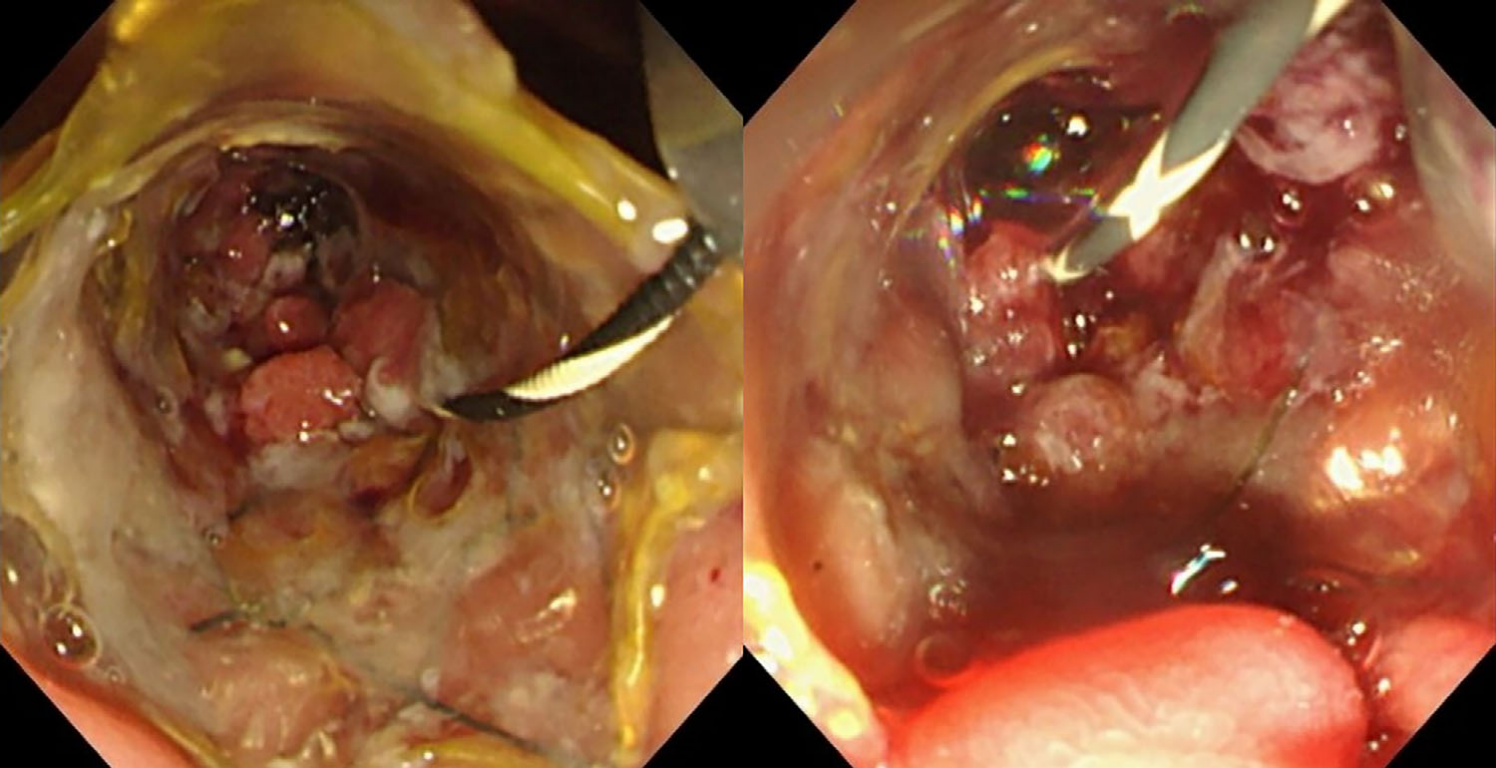
Endoscopic images of small tissue ingrowths that form in the multiple small side holes of multi‐hole self‐expandable metal stents. The images were taken sixty‐eight days after stent placement.

A recent retrospective study^8^ comparing MHSEMS with c‐CMS and uncovered SEMS showed that tumor ingrowth occurred most commonly in the uncovered SEMS group (13.2% vs. 0% vs. 42.4% for MHSEMS, c‐CMS, and uncovered SEMS, respectively) while stent migration occurred most commonly in the c‐CMS group (2.6% vs. 15.8% vs. 0% for MHSEMS, c‐CMS, and uncovered SEMS, respectively), resulting in the lowest RBO rate (21% vs. 37% vs. 55% for MHSEMS, c‐CMS, and uncovered SEMS, respectively) and the longest mean TRBO (479 vs. 353 vs. 306 days for MHSEMS, c‐CMS, and uncovered SEMS, respectively) in the MHSEMS group. However, several issues must be considered when interpreting the results of the study. First, the RBO rate and TRBO of MHSEMSs were not significantly different when compared to c‐CMSs. Second, many cases in the MHSEMS and c‐CMS groups were censored early on after SEMS placement, potentially affecting the RBO rate and mean TRBO. Finally, several types of stents were used in each group. In our study, the main body designs of the two stents were exactly the same, enabling an accurate evaluation of the impact of the addition of multiple side holes on the prevention of stent migration and TRBO. As expected, both tumor ingrowth and stent migration were not observed in this study, which rates were quite low compared to the results of the previous study (tumor ingrowth: 13.2%; stent migration: 2.6%).^8^ The discrepancies between the two studies may be in part due to differences in the number and location of side holes and the body designs of the stents. In fact, the rate of tumor ingrowth was reported to be lower when the number of side holes was reduced (31% vs. 4%).^8^ Although the rate of stent migration was significantly lower in the MHSEMS group (0% vs. 17.6%, *p* = 0.032), occlusion (30.8% vs. 26.5%, *p* = 0.78) and non‐occlusion cholangitis (23.1% vs. 11.8%, *p* = 0.31) were more common in the MHSEMS group, resulting in similar overall RBO rates between the two groups. The relatively higher rate of RBO due to stent occlusion and non‐occlusion cholangitis might be explained by differences in the mechanical properties of the two stents. Although the body designs of the two stents were identical, the radial force of MHSEMS might be lower as a result of adding side holes on the covering membrane. Indeed, the diameter of SEMS at the level of the biliary stricture was smaller in those who received MHSEMS (5.6 mm vs. 6.2 mm, *p* = 0.10). Multiple small ingrowths in the common bile duct may hamper bile flow (including antegrade flow of duodenobiliary reflux), potentially contributing to stent dysfunction (Figure 3). This may be an important issue considering that duodenobiliary reflux is very common when SEMSs are placed across the papilla.^14^ Furthermore, cholestasis may occur more frequently with MHSEMS compared to uncovered SEMS due to the uneven surface of ingrowths.

As the prognosis of PC has improved due to recent advances in chemotherapy, ERI after RBO of the initial SEMS is becoming more and more important. Although stent removal of MHSEMS was successful in all attempted cases in this study with a median indwelling period of 20 (range, 3–122) days, there are concerns about whether MHSEMSs can be safely removed, especially after long indwelling periods. Bleeding after stent removal is another concern, which led us to avoid stent removal in a patient experiencing hemorrhage from the tumor. Outcomes after ERI were not significantly different between the two groups, with high technical and clinical success rates. Conversion to EUS‐guided intervention is another salvage method after RBO of MHSEMSs, particularly when the stent cannot be removed or when transpapillary biliary drainage is not feasible due to duodenal obstruction.

We acknowledge several limitations of our study. This was a retrospective study from a single institution with inevitable selection bias. Prophylactic rectal NSAID use tended to be different between the two groups (*p* = 0.061), which may have affected the rate of pancreatitis.

In conclusion, MHSEMS was associated with a significantly shorter TRBO compared to c‐CMS in SEMS‐naïve patients. Further modifications of the present MHSEMS, including the size and number of side holes and body designs of the stent, may be needed before using this stent as first‐line biliary drainage for DMBO in unresectable PC.

## CONFLICT OF INTEREST STATEMENT

None.

## ETHICS STATEMENT

Approval of the research protocol by an Institutional Reviewer Board: This study was conducted in accordance with the master protocol (2023‐GB‐077) for gastrointestinal, hepatic, and pancreatobiliary diseases, which was approved by the institutional review board of our institution.

## PATIENT CONSENT STATEMENT

Informed consent for this study was waived by the institutional review board owing to the retrospective study design.
